# Stable Isotope Tracking of Endangered Sea Turtles: Validation with Satellite Telemetry and δ^15^N Analysis of Amino Acids

**DOI:** 10.1371/journal.pone.0037403

**Published:** 2012-05-29

**Authors:** Jeffrey A. Seminoff, Scott R. Benson, Karen E. Arthur, Tomoharu Eguchi, Peter H. Dutton, Ricardo F. Tapilatu, Brian N. Popp

**Affiliations:** 1 National Oceanic and Atmospheric Administration–National Marine Fisheries Service, Southwest Fisheries Science Center, La Jolla, California, United States of America; 2 Department of Geology and Geophysics, University of Hawai’i at Manoa, Honolulu, Hawai’i, United States of America; 3 Marine Laboratory, The State University of Papua, Manokwari, Papua Barat Province, Indonesia; 4 Department of Biology, University of Alabama, Birmingham, Alabama, United States of America; Monash University, Australia

## Abstract

Effective conservation strategies for highly migratory species must incorporate information about long-distance movements and locations of high-use foraging areas. However, the inherent challenges of directly monitoring these factors call for creative research approaches and innovative application of existing tools. Highly migratory marine species, such as marine turtles, regularly travel hundreds or thousands of kilometers between breeding and feeding areas, but identification of migratory routes and habitat use patterns remains elusive. Here we use satellite telemetry in combination with compound-specific isotope analysis of amino acids to confirm that insights from bulk tissue stable isotope analysis can reveal divergent migratory strategies and within-population segregation of foraging groups of critically endangered leatherback sea turtles (*Dermochelys coriacea*) across the Pacific Ocean. Among the 78 turtles studied, we found a distinct dichotomy in δ^15^N values of bulk skin, with distinct “low δ^15^N” and “high δ^15^N” groups. δ^15^N analysis of amino acids confirmed that this disparity resulted from isotopic differences at the base of the food chain and not from differences in trophic position between the two groups. Satellite tracking of 13 individuals indicated that their bulk skin δ^15^N value was linked to the particular foraging region of each turtle. These findings confirm that prevailing marine isoscapes of foraging areas can be reflected in the isotopic compositions of marine turtle body tissues sampled at nesting beaches. We use a Bayesian mixture model to show that between 82 and 100% of the 78 skin-sampled turtles could be assigned with confidence to either the eastern Pacific or western Pacific, with 33 to 66% of all turtles foraging in the eastern Pacific. Our forensic approach validates the use of stable isotopes to depict leatherback turtle movements over broad spatial ranges and is timely for establishing wise conservation efforts in light of this species’ imminent risk of extinction in the Pacific.

## Introduction

Elucidating patterns of migratory connectivity for broad-ranging animals is central to defining spatio-temporal management priorities for these species. Knowledge about movements and foraging area destinations can discern the relative value of discrete regions for resource acquisition, and when examined at a population level, can provide insights into demographic consequences of habitat selection patterns [1]–[3]. However, inherent challenges of directly observing long-distance migratory behaviors often require creative application of existing tools to infer prevailing movement patterns [4], [5].

Satellite telemetry has been a primary tool to track long-distance movements of vertebrates from around the world [1], [6], [7], but the high cost of tags, satellite tracking data acquisition, and analysis (up to US $5K per animal) coupled with large body size requirements for tracked animals have precluded its application for many vulnerable species and age classes. These challenges have often resulted in extremely limited samples sizes, which thereby limit the value of telemetry as a tool for revealing migratory variability within focal populations. A new approach to rapidly assess the movements of large cross sections of imperiled populations could greatly enhance conservation of such animals.

Stable isotope analysis (SIA) is a relatively low cost, complementary approach to satellite tracking of animal movements. Isotopic compositions of consumer tissues integrate information from foraging environments [8], and thus, when an animal moves among spatially discrete food webs that are isotopically distinct (often termed ‘isoscapes’), stable isotope values of its tissues can provide unambiguous information about its previous location [4], [5], [9]–[14]. An additional advantage is that the stable isotope tracking method does not require initial marking of individuals, and thus every ‘capture’ (i.e. sampled animal) provides information on prior whereabouts.

Stable isotope animal tracking has been employed successfully for a variety of terrestrial and aerial species, due in large part to the well-understood systematic patterns of stable isotope variations in meteoric water and plants across continents [5], [13], [14]. In marine systems, however, patterns of isotopic abundances are poorly resolved for most ocean regions. Spatial gradients in stable isotope values at the base of the food web have been described on very large scales [15], [16], but few regional maps are available [17] and little is known about temporal shifts in isotope abundances in natural systems [17], [18]. For high-order consumers, the elaboration of foraging habitats is further obscured by the substantial difference in stable isotope values between their tissues and those of basal producers [8], [18]–[20]. Clearly, isotopic tracking of marine species would benefit from a greater understanding of spatiotemporal isotopic patterns in marine fauna as well as from clarification about the baseline influence on isotopic compositions of tissues of higher-order marine consumers [21].

Integrative studies that combine satellite tracking with stable isotope analysis are needed to validate the isotope tracking method [20], [22]. However, confounding such studies is the inability of bulk tissue analysis to distinguish the influence of a consumer’s trophic status from the effect of baseline (i.e. primary producer) isotope values of the ecosystem within which that animal resides [22], [23]. The application of compound-specific isotopic analyses of amino acids (CSIA-AA) can elucidate the differential impacts of these factors and thus can substantially enhance the value of stable isotope measurements for tracking marine species [21], [24]. This is possible because some AAs, such as phenylalanine, retain the isotopic composition of source nitrogen at the base of the food web, whereas other AAs, such as glutamic acid, are significantly enriched in ^15^N as they move through the food web [25], [26]. Baseline and trophic information can therefore be obtained from a consumer’s tissue without need for analyses of prey items or of basal food web samples [24], [26]. To date, however, this technique has not been validated for most taxa [27].

Marine turtles are an ideal taxon for such integrative studies due to their broad ranging movements, residence in discrete foraging areas, and fidelity to specific nesting beaches, where they can be easily sampled and equipped with telemetry devices [6]. Further, the extremely slow isotopic replenishment rates (i.e. turnover) of soft tissues of turtles [28], [29] allows for prevailing isotope regimes at foraging areas to be traced long after a subject’s departure. Leatherback turtles (*Dermochelys coriacea*), in particular, are highly mobile with migrations often spanning entire ocean basins [3]. They forage exclusively on gelatinous prey such as scyphomedusae and pyrosomes [30]–[33] that commonly show isotopic values that reflect regional food webs [34], [35]. Supported by previous studies that confirm the value of SIA for studying their life history [30], [36]–[38], leatherbacks are an ideal species in which to explore the value of stable isotope tracking to depict foraging areas over broad scales.

Pacific leatherbacks are considered at imminent risk of extinction [39] due to long-term harvest of eggs and adults and incidental capture in fishing gear [3], [39]. Knowledge of foraging areas and migratory connectivity in the Pacific, where these threats persist, is critical to the sustained management of leatherbacks. Satellite telemetry efforts have shown that leatherbacks nesting in Papua Barat Province, Indonesia inhabit multiple foraging areas throughout the Pacific [40]. However, despite these insights there is no information about the extent to which an individual’s putative foraging area influences the isotopic compositions of its body tissues, nor is their information about patterns of individual philopatry to specific foraging areas. If adequately resolved, the isotope tracking method may provide a means to determine foraging regions for a large number of Pacific leatherbacks and thus provide information vital to the development of effective management strategies.

Here, we build upon previous satellite tracking efforts [40] and examine the migratory strategies of Indonesian leatherbacks using nitrogen isotopic compositions of bulk tissue, the results of which are validated with compound specific nitrogen isotopic analysis of amino acids and satellite telemetry. Because of the apparent variability in nitrogen isotope abundances in the eastern vs. western Pacific [15], [16], and the fact that individuals from this stock forage in both broadly separated regions [40], we hypothesized that the stable nitrogen isotope ratios in leatherback skin tissue collected at the Indonesian nesting beach would reflect the foraging region used by individual turtles prior to the nesting season. If δ^15^N values of the tissues of turtles from these separate regions are sufficiently different, our approach may foster a transition from satellite telemetry to stable isotope tracking as a means to decipher animal movements in the Pacific and across ocean basins in general.

## Results

**Figure 1:**
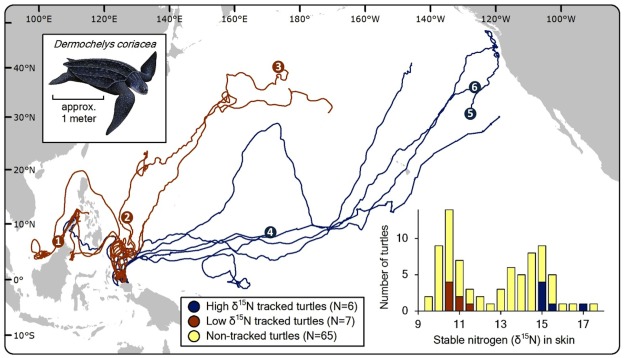
Map of satellite-tracked post-nesting movements of 13 leatherback turtles from Jamursba-Medi, Papua Barat Province Indonesia, overlaid with stable nitrogen isotopic values for skin tissue of nesting females from the same population. Satellite-tracked turtles were studied in 2007 and 2010; non-tracked turtles (*n* = 65) were skin sampled in 2005–2007 and 2010. Blue and red track lines depict turtles within the ‘high δ^15^N’ and ‘low δ^15^N’ groups, respectively. Tracks with numbered termini depict movements of leatherbacks that were analysed for CSIA-AA (Table 2).

Leatherbacks that were satellite tracked in this study departed their Indonesian nesting beach *en route* to two broad regions (Figure 1): (i) the ‘western Pacific’ (*n* = 8), with primary destinations in the Sulawesi, Sulu, and South China Sea region and the North Pacific Transitional Zone (NPTZ), and (ii) the ‘eastern Pacific’ (*n* = 5), with the prevailing terminus centered in the California Current Large Marine Ecosystem. Tracking data were acquired for an average 312±131 days per turtle, with six turtles tracked for more than one year (maximum tracking duration  = 519 days; Table S1). Most turtles, but not all, were tracked long enough to distinguish their particular foraging area. Putative foraging regions were corroborated by results from previous tracking efforts [40] that applied state-space models to show that the majority of these turtles had commenced area-restricted search behavior (i.e., assumed foraging) by the end of their respective tracking. There were two abbreviated tracks, with durations of 46 d and 95 d; however, the orientation of departure for both turtles, coupled with the consistency of this departure trajectory among numerous other turtles tracked to the western Pacific [40] indicated they were moving to western Pacific foraging grounds.

**Figure 2:**
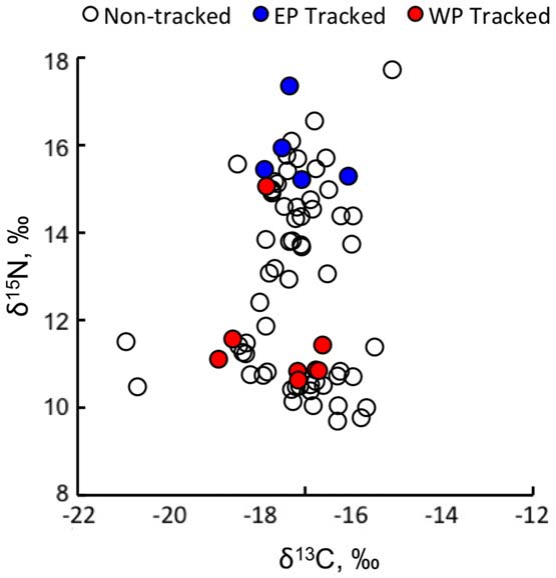
Scatterplot of stable carbon vs. stable nitrogen values for 78 leatherback turtles sampled at the Jamursba-Medi nesting beach in Papua Barat Indonesia.

**Figure 3:**
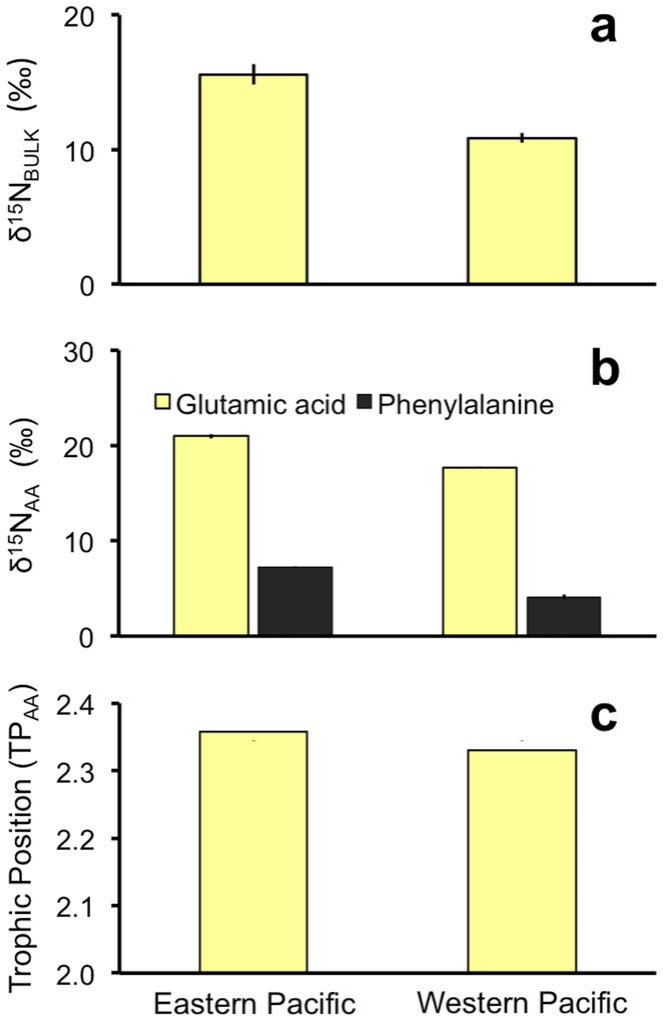
Leatherback turtle skin nitrogen stable isotopic compositions. **a**, nitrogen isotopic composition of skin from leatherback turtles known to feed in the eastern and western Pacific; **b**, nitrogen isotopic composition of glutamic acid and phenylalanine in skin tissue from leatherback turtles feeding in the eastern and western Pacific; **c**, trophic position calculated using the isotopic compositions of glutamic acid and phenylalanine [17], presented as a weighted average based on AA measurement error. Error bars represent weighted standard deviation of trophic position means; *n* = 3 each. While there was a significant difference in skin δ^15^N between eastern and western Pacific foragers (t-test: *p*<0001), there was no significant difference in trophic position between these two groups (t-test: *p* = 0.682).

Leatherback epidermis (stratum corneum, hereafter referred to as ‘skin’) samples (*n* = 78) had δ^13^C values ranging from –20.9 ‰ to –15.1 ‰ and δ^15^N values from 9.7 ‰ to 17.7 ‰ (Table 1, Figure 2). Bulk skin δ^13^C values were normally distributed about a single mean (–17.3±1.0 ‰), whereas bulk skin δ^15^N values had a strongly bimodal distribution with distinct ‘low δ^15^N’ (range = 9.7–12.4 ‰, *n* = 36) and ‘high δ^15^N’ (range = 12.9–17.7 ‰, *n* = 42) groups (see inset Figure 1, Figure 3a). When linked with telemetry results, we found that all turtles tracked to the eastern Pacific were within the high-δ^15^N group, whereas all but one turtle moving to western Pacific sites were in the low-δ^15^N group (Figure 1). However, despite the apparent spatial influences on δ^15^N values, these approaches alone could not rule out trophic influences on the measured δ^15^N disparity.

**Table 1:** Summary of leatherback tissue sampling and δ^13^C and δ^15^N values for turtles nesting in Jamursba Medi, Papua Barat, Indonesia.

Year	No. Turtles	Sample date (dd/mm/yy)	δ^ 13^C range (‰)	δ^15^N range (‰)
1	39	03/07/05 – 09/08/05	–20.9 to –15.1	9.7 to 17.7
2	21	24/06/06 – 27/07/06	–20.7 to –15.5	10.0 to 16.5
3	14	22/07/07 – 29/07/07	–18.9 to –16.6	10.6 to 17.3
4	4	09/06/10 – 11/06/10	–18.5 to –16.0	10.4 to 15.3
Overall	78	03/07/05 – 11/06/10	–20.9 to –15.1	9.7 to 17.7

Satellite tags were applied to 12 of 14 turtles sampled 2007 and one of four sampled in 2010.

Using CSIA of phenylalanine (a ‘source’ AA, *sensu* ^16^), we found average δ^15^N_phenlyalanine_ (± SD) values of 7.27±0.03‰ and 4.17±0.05‰ for eastern and western Pacific foragers, respectively (Figure 3b). Combining δ^15^N_phenlyalanine_ values with δ^15^N of glutamic acid from the same samples (Table 2, Figure 3b) indicated that there was no difference in the trophic position for these two foraging groups (weighted average TP_EP_ ± s.d.  = 2.36±0.01, TP_WP_ ± s.d.  = 2.35±0.01, *P* = 0.682; Figure 3c), and that the difference in bulk skin δ^15^N values was instead driven by the differences in baseline nitrogen isotopic composition between the two regions.

**Table 2:** δ^15^N values (‰) of source and trophic amino acids in leatherback turtles known from satellite tracking to migrate to the western and eastern Pacific Ocean.

	*Western Pacific*			*Eastern Pacific*			
Compound	Turtle 1	Turtle 2	Turtle 3	Turtle 4	Turtle 5	Turtle 6	
*Trophic AAs*							
Alanine	20.9±0.5	18.4±0.4	21.2±0.5	24.5±0.4	21.4±0.6	24.0±0.1	
Valine	19.2±0.5	19.4±0.2	21.1±0.1	22.1±0.1	18.7±0.5	21.5±0.5	
Leucine	17.2±0.5	18.6±0.1	18.7±0.6	21.9±0.9	19.7±0.5	21.8±0.5	
Isoleucine	17.2±0.3	18.0±0.7	18.2±0.4	21.5±0.1	18.2±0.1	21.0±0.1	
Aspartic Acid	13.6±0.2	14.0±0.3	14.6±0.3	17.7±0.8	15.8±1.0	17.4±0.1	
Glutamic Acid	17.4±0.4	17.4±0.3	18.1±0.2	21.3±0.6	20.2±0.7	21.4±0.3	
	Average Trophic AA = 18.0± *0.6 ‰			Average Trophic AA = 20.6± *1.4 ‰			
*Eastern Pacific – Western Pacific Trophic AA = 2.6 ‰*							
*Source AAs*							
Glycine	10.2±0.1	13.2±0.7	9.4±0.3	16.2±0.1	11.9±0.8	14.7±0.3	
Phenylalanine	4.9±0.7	3.6±0.5	4.0±0.3	7.4±0.4	6.9±0.3	7.5±0.4	
Serine	8.5±0.3	9.5±0.1	7.5±0.6	13.5±0.2	12.6±0.3	12.0±0.2	
Tyrosine	4.9±0.6	4.2±0.4	3.1±0.6	7.2±0.4	6.4±0.6	7.6±0.6	
	Average Source AA = 6.9± *0.6 ‰			Average Source AA = 10.3± *0.6 ‰			
*Eastern Pacific – Western Pacific Source AA = 3.4 ‰*							
*Trophic Position (TP)*							
	2.2±0.2	2.4±0.2	2.4±0.1	2.4±0.2	2.3±0.2		2.3±0.2
	*Average TP, Western Pac. = 2.36±0.01*			*Average TP, Eastern Pac. = 2.35±0.01*			

*
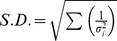

**Figure 4:**
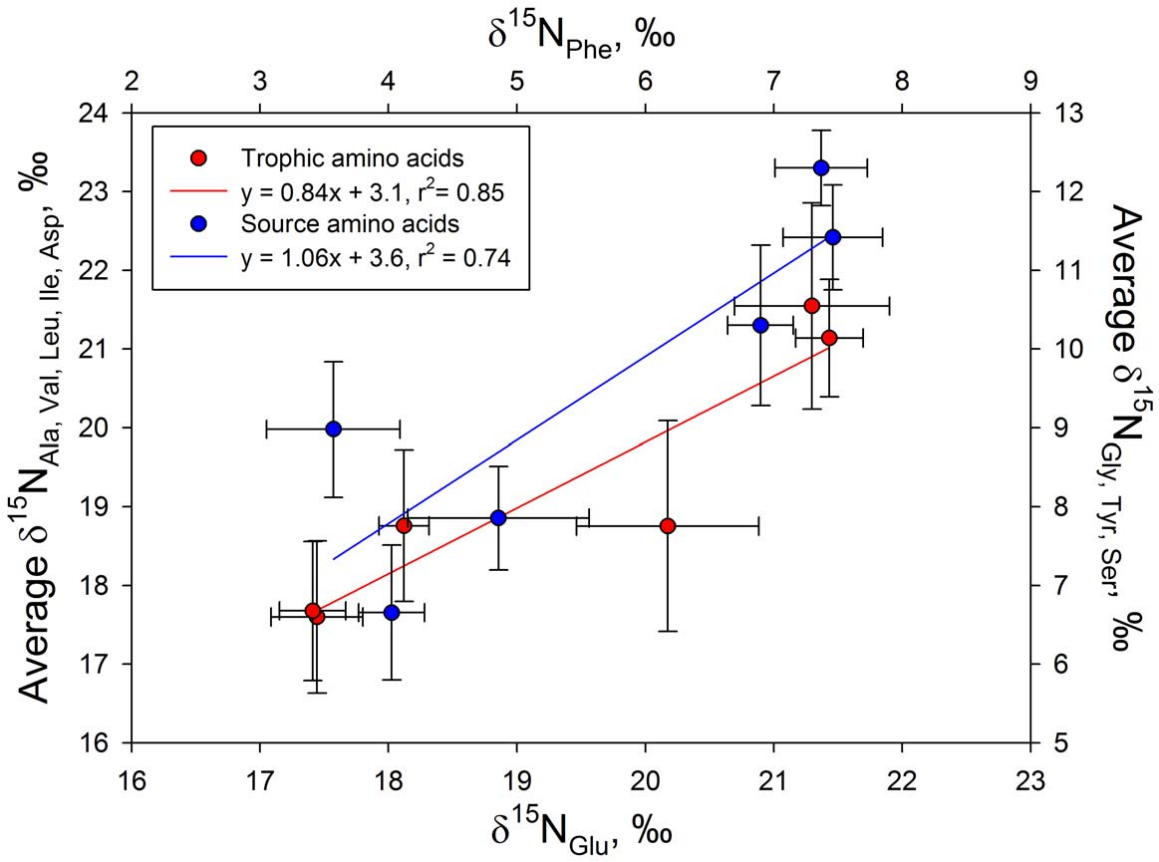
Plot of the δ^15^N value of glutamic acid versus the average δ^15^N value of the trophic amino acids alanine, valine, leucine, isoleucine and aspartic acid (red), and the δ^15^N value of phenylalanine versus the average δ^15^N value of the source amino acids glycine, tyrosine and serine (blue). The slopes of the regression lines in this figure are not different at the 95% confidence interval. The satellite-tracked movements of each turtle are shown in Figure 1.

We also examined the nitrogen isotopic compositions of a wide array of other trophic (i.e., alanine, valine, leucine, isoleucine and aspartic acid) and source (i.e., glycine, tryosine and serine) amino acids to determine if they too were consistent with interpretation of the results from only glutamic acid and phenylalanine as per previous marine ecosystem studies using CSIA-AA [41]. The δ^15^N_glutamic acid_ values were highly correlated with the average δ^15^N values of the trophic amino acids and the δ^15^N_phenlyalanine_ values were highly correlated with the average δ^15^N values of the source amino acids (Figure 4). In addition, the average differences in the δ^15^N values of eastern and western Pacific trophic and source amino acids averaged ca. 3 ‰, which is similar to the difference in means between the bulk skin high-δ^15^N and low-δ^15^N groups (Table 2).

**Figure 5:**
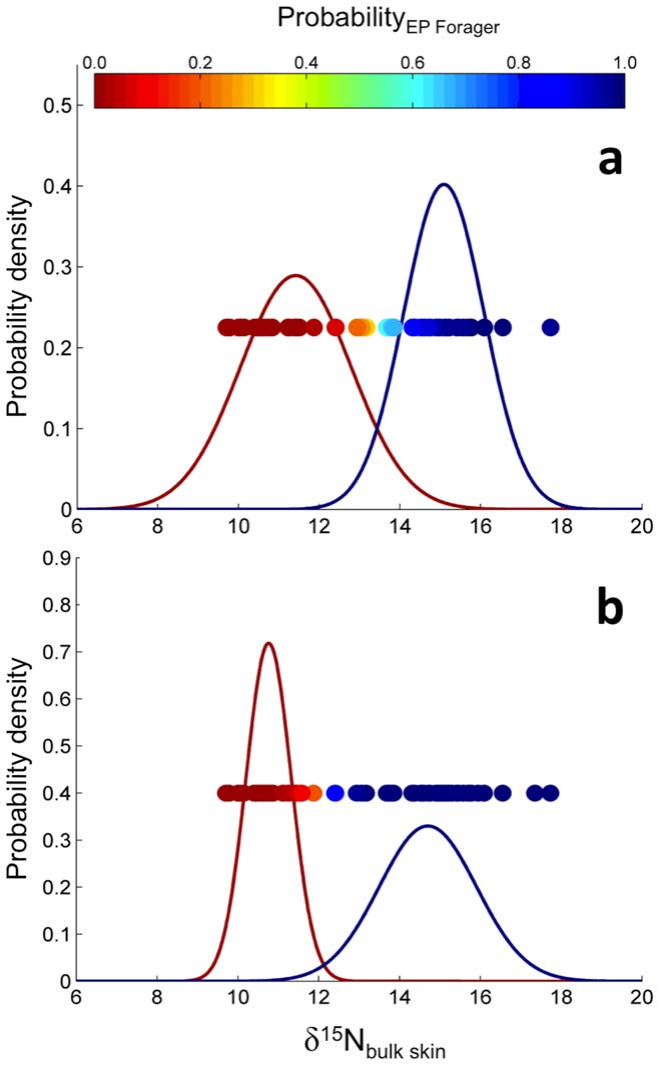
Marginal posterior distributions of δ^15^N values for the two assumed foraging grounds (bell-shaped curves) and individual assignments to one of the two foraging grounds (filled circles in horizontal arrangement) with color-coded probabilities of belonging to the eastern Pacific foraging area. Panel A is from Model 4, whereas the Panel B is from Model 6. For both models, posterior distributions depicted in red are from the ‘low δ^15^N’ (i.e. putative western Pacific foragers), whereas those in blue represent the ‘high δ^15^N’ group (i.e., putative eastern Pacific foragers).

The disparate bulk skin δ^15^N values between known (i.e. satellite-tracked) eastern and western Pacific foragers combined with CSIA-corroboration that these differences were due to baseline isotope values in each region allowed us to characterize the foraging area destinations for each individual based on its skin δ^15^N value using a Bayesian mixture-model analysis. Among the six models considered (Table 3), all models performed well and no model stood out as the best according to our model selection method. Models 1 through 4 performed well in terms of the mean of the differences (near zero - unbiased), but Models 5 and 6 performed well with respect to the variability in the differences (good precision). Estimated variances of the two normal distributions were different (Figure 5), indicating two-variance models (Models 2, 4, and 6), were more appropriate than the one-variance models (Models 1, 3, and 5). One turtle (72489; Table S1) exhibited an eastern Pacific δ^15^N value (i.e. ‘high’ δ^15^N: 15.05‰) but was tracked to the western Pacific (Figure 1). Consequently, the results of the models that used the known foraging locations were influenced by this turtle, and resulted in more-precautionary foraging region assignments. Because of the likelihood that the proportion of foraging destinations changes annually, we use Model 4 (known foraging destinations, time-independent) and Model 6 (unknown foraging destinations, time-dependent) (Table 3) to assign foraging region to each turtle.

**Table 3:** Definitions of four models used to estimate the underlying isotopic signatures of the Eastern and Western Pacific Ocean and to assign each individual to one of the two foraging grounds.

Model	Foraging ground isotopic signatures	Treatments of telemetry data	Proportion of foraging destinations
1	one variance	unknown	Time independent
2	two variances	unknown	Time independent
3	one variance	known	Time independent
4	two variances	known	Time independent
5	one variance	unknown	Time dependent
6	two variances	unknown	Time dependent

The two estimated distributions of δ^15^N values (Figure 5) indicated that there were two isotopically distinct foraging regions for these turtles. All turtles that were tracked to the eastern Pacific were assigned to the distribution with the higher mean, whereas all but one turtle (72489; Table S1) that were tracked to the western Pacific foraging area were assigned to the distribution with the lower mean (Figure 1). Results of Model 4 included nine ambiguous assignments using our cut off point of 0.8 (Table 4); these nine turtles had intermediate skin isotopic compositions and thus could not be assigned to either region. Model 6, which did not incorporate known foraging areas, however, assigned all turtles to one of the two foraging grounds with probability greater than 0.8 (Table 4).

**Table 4:** Parameter estimates and results of mixture model assignments for eastern Pacific (EP) and western Pacific (WP) foraging.

	Model 4	Model 6
**Median δ^15^N (EP vs. WP)**	15.09 vs. 11.42	14.70 vs. 10.76
	[14.64, 15.55] vs. [10.91, 12.19]	[14.24, 15.08] vs. [10.55, 10.98]
**SD δ^15^N (EP vs. WP)**	0.99 vs. 1.38	1.21 vs. 0.56
	[0.73, 1.37] vs. [1.02, 1.98]	[0.95, 1.64] vs. [0.43, 0.77]
**# assigned (EP vs. WP) [total]**	2005: 14 vs. 17 [39]	2005: 13 vs. 26 [39]
	2006: 14 vs. 6 [21]	2006: 13 vs. 8 [21]
	2007: 0 vs. 1 [1]	2007: 7 vs. 7 [14]
	2010: 2 vs. 1 [3]	2010: 1 vs. 3 [4]
**% EP Foragers**	0.57 [0.43, 0.74]	2005: 0.33 [0.20, 0.49]
		2006: 0.60 [0.39, 0.79]
		2007: 0.49 [0.25, 0.73]
		2010: 0.34 [0.06, 0.77]

Model 4 used known foraging areas so that fewer individuals were assigned using the model results. Model 6 did not use known foraging areas and assigned all turtles to one or the other foraging area. Values are medians and 95% probability intervals are in brackets.

The proportion of turtles that foraged in the eastern Pacific was estimated from Model 4 to be approximately 0.57 annually (median, 95% posterior interval: 0.43–0.74). Using Model 6, which did not use the telemetry data, the annual proportion of turtles that use the eastern Pacific ranged from 0.3 to 0.6, although the small sample sizes resulted in large uncertainty (Table 4).

## Discussion

Here we present the first study integrating satellite tracking with stable isotope analysis of relevant bulk tissues and amino acids to study marine turtle movements and stable isotope ecology. Isotope spatial gradients have been proposed as a viable means to track disparate foraging groups of marine turtles [42], [43] and seabirds [22], [23]; however, these studies were unable to decipher the influence of baseline control (i.e. spatial effect) vs trophic variation on isotope values. By integrating the CSIA approach, we demonstrate that differences in δ^15^N values of bulk skin from leatherbacks are not the result of variation in trophic position between groups, but instead due to differences in the baseline values of primary producers between two broad regions in the Pacific. Although control on tissue δ^15^N values of marine animals by the biogeochemistry of a region has been demonstrated based on AA-CSIA on local and global scales for other organisms [24], [44], it has not been demonstrated in marine turtles or other highly migratory species, nor has this observation been corroborated via satellite telemetry.

The application of CSIA to a broad group of trophic and source amino acids indicated that leatherbacks from the eastern and western Pacific foraged at virtually identical trophic levels (TP_AA_ ∼2.4 based on the δ^15^N values of glutamic acid and phenylalanine; Figure 3c). This is a rather low trophic position for a large marine mega-vertebrate, yet not surprising considering that leatherback turtles consume gelatinous prey [3], [32], [33]. In the eastern Pacific, leatherbacks forage largely on the sea nettle (*Chrysaora fuscescens*) [40, S. Benson, unpubl. data], but in the western Pacific their diet is less clear, although there too leatherbacks are known to forage on gelatinous prey [33]. Additional empirical dietary data on leatherbacks would help clarify the precision of CSIA-AA for establishing a species’ trophic status. Regardless, the similarity in trophic status between leatherbacks foraging in the two ocean regions as determined by the isotopic compositions of a wide array of trophic and source amino acids signals an important step forward in our understanding of the isotope ecology of this critically endangered species.

The fact that leatherbacks are believed to inhabit foraging regions for the entirety of their non-breeding life phases [2], [40] suggests that the isotope profiles of these regions were able to fully integrate into leatherback body tissues prior to the commencement of their return migration to the nesting beach. However, it is likely that some individuals access multiple spatially disparate and ephemeral foraging sites within a broad region during non-reproductive periods. In the Atlantic, for example, leatherbacks may forage in the northeastern Atlantic during the first year of their migration and then cross the Atlantic to forage along the Canadian coasts or vice versa [45], [46]: leatherbacks are also known to undertake extensive seasonal north-south movements in the western North Atlantic during non-breeding periods [47]. Indeed among tracked leatherback turtles in the Pacific, leatherbacks have been shown to forage along the central California coast during late summer and fall, and move into more tropical latitudes of the eastern Pacific during winter periods [40]. In the Pacific, however, there are no data indicating that a leatherback would forage both in the eastern and western Pacific during a single inter-breeding cycle [40]. Thus, considering the elevated δ^15^N values throughout the eastern Pacific due to influences of denitrification, we believe that even if a turtle accessed multiple foraging hotspots in the region, prey in these areas would all have elevated δ^15^N values.

Isotopic turnover in adult turtle skin is believed to be on the order of four to six months [35], [36], and thus longer than most pre-nesting, foraging area-to-nesting beach migratory durations of Indonesian leatherbacks [40]. This suggests that upon arrival at the nesting beach, the δ^15^N of leatherback skin continues to reflect the isotopic conditions in the distant foraging areas, with the isotopic values most closely reflecting conditions at the last foraging site accessed by a turtle prior to the nesting migration. Further, even for the longest of migratory durations, the skin δ^15^N values are largely, if not exclusively, derived from foods consumed at distant foraging areas because marine turtles generally do not forage during migratory movements [3], unlike other taxa such as seabirds [48].

Interestingly, we found little difference in δ^13^C values of bulk skin between putative eastern and western Pacific foragers. This was unexpected considering that carbon isotope values of tissues have been used to track a variety of marine animals across large ocean regions [20], [48], [49]. Lack of bulk skin δ^13^C variability suggests that carbon isoscapes are more uniform in areas where leatherbacks foraged, despite the well-documented oceanic-neritic gradient in δ^13^C values of marine primary producers [50]. Perhaps the lesser magnitude of δ^13^C variation is due to the fact that leatherbacks foraged primarily in coastal regions in both eastern and western Pacific foraging grounds, with less use of offshore regions [2], [3], [40]. Nevertheless, we would expect turtles foraging in the relatively eutrophic waters of the eastern Pacific to diverge in their skin δ^13^C values from those turtles foraging in the shallower, oligotrophic waters of the Sulu Suluwesi and South China Sea regions due to the disparate oceanography of these two broad regions [51]. Although the reason(s) for low δ^13^C variability are unclear, our data indicate that nitrogen isotope values of skin are more effective than carbon for tracking the broad-scale movements of leatherback turtles, and perhaps other high seas-dwelling vertebrates in the Pacific. To further resolve spatial patterns in δ^13^C we encourage additional isotopic studies that focus on satellite-tracked Pacific leatherbacks.

The nitrogen isotope dichotomy in leatherback skin δ^15^N values was likely due to differences in nitrogen biogeochemistry in the eastern versus western Pacific [15]. The influence of nitrogen cycling on the δ^15^N values in marine turtle tissues has been suggested previously [34], [52]; however, so far no studies have examined δ^15^N in tissues of turtles from two N cycling regimes in the same ocean basin. In the present study, leatherbacks tracked to the western Pacific remained in an area dominated by N_2_-fixation, where source nitrogen has a lower isotopic composition [16], [53]. Turtles moving to the eastern Pacific – presumably to take advantage of gelatinous prey blooms in the California Current Large Marine Ecosystem [40] – enter a region characterized by high phytoplankton δ^15^N values in surface waters [54] caused by denitrification in the eastern Tropical Pacific [15], [55] and advection of this water mass northward [56]. Significantly, leatherbacks nesting in Pacific Costa Rica, all of which remain in the eastern Pacific for their entire post-nesting migrations [2], did not show bimodality in δ^15^N values of nesters, and instead had bulk tissue δ^15^N values more in line with our eastern Pacific group (mean δ^15^N_yolk_  = 14.7±2.0 ‰ [37]), underscoring the uniformity in δ^15^N values among leatherbacks foraging throughout the eastern Pacific, and the role of nitrogen cycling regimes in driving observed patterns of marine turtle nitrogen isotopic compositions.

We acknowledge that the nitrogen isoscape spatial heterogeneity is likely more complex than the bimodal ‘eastern/western’ scenario present here. Indeed, previous simulations of δ^15^N patterns in surface waters of the Pacific based on N cycling regimes [15], [16], [57] revealed high-δ^15^N hotspots in the mid latitudes of the northeastern and southeastern Pacific, along with areas of low predicted δ^15^N spanning much of the North Pacific. Although empirical studies of δ^15^N values of baseline producers collected *in-situ* are needed, these models underscore the variability in δ^15^N values of surface waters across the Pacific. Two factors likely contributed to the dichotomous δ^15^N results. First, despite the long distance of satellite tracks, leatherback movements were restricted to relatively few areas of the Pacific, perhaps reflecting only a subset of all Pacific N isoscapes. For example, based on extensive previous satellite tracking efforts [40] it is probable that any east-bound turtle ultimately moved into the same general area along the Pacific coast of North America (central California USA to British Columbia Canada) to forage. Thus, most if not all eastern Pacific foragers likely integrated the composition of an isotopically uniform region. For those remaining in the western Pacific, there was more variation in foraging destination, with turtles moving to the North Pacific Transitional Zone or to the Indo-Pacific regions of the South China and Sulu Suluwesi seas. Yet despite the great distances that separate these areas, the entire region is characterized by a relatively uniform N cycling regime, with N-fixation as the dominant process [15], [16], [57]. Second, the bimodality in δ^15^N values of leatherback skin may result from the inability of skin - a slow turnover tissue - to capture the variability of δ^15^N as turtles move through different nitrogen isoscapes during foraging and reproductive migrations. Had a faster turning over tissue such as blood plasma been studied, perhaps a more complex isoscape would have been observed. However, such a tissue would likely have turned over too fast, thereby precluding the conservation of foraging region δ^15^N influences during the longest of nesting migrations [40].

Considering the integrative nature of this study, a primary caveat is that the relationship between leatherback skin δ^15^N values and satellite tracking destinations is partially hindered because isotopic compositions provide information on foraging area before nesting and satellite tracking pinpoints areas used after nesting. Effectively, these two techniques have a time lag between them. However, the fact that our Bayesian foraging area assignment tests (Model 6) correctly predicted the foraging destination of 12 of 13 (92%) of the tracked turtles underscores the value of the multi-tool approach despite this temporal disconnect. Nevertheless, to alleviate this issue in the future, we recommend that turtles are equipped with transmitters in foraging areas and tracked to nesting beaches, thus the satellite track and nesting beach skin sample would be reflective of the same time period. Further, if at the time of tagging, a tissue sample was collected, we could better understand the changes in isotopic composition during a nesting migration [*sensu* 58].

### Conservation Value of Leatherback Isotope Tracking

Our forensic approach provides a solution to identifying spatial use patterns across vast areas for vulnerable, migratory marine species, and signals a promising, cost-effective technique for determining the spatial affinities of other highly migratory marine animals. We agree that the spatial resolution of the isotope tracking approach is orders of magnitude less than that of satellite tracking. However, satellite telemetry may cost up to US$ 5K per tracked animal, whereas the bulk tissue SIA and CSIA-AA for the same animal together cost < US$ 200. The substantial savings underscores the potential for examining a large number of individuals from a population of interest. It would also be instructive to employ additional cost-effective analysis of biomarkers such as trace elements [59] to determine if greater spatial resolution of leatherback migratory strategy can be gained by a larger suite of biogeochemical approaches.

Despite the apparent lesser spatial resolution afforded by stable isotope tracking, this technique has important conservation value for leatherback turtles. For example, the concordance of individual turtles’ δ^15^N groupings with distinct post-nesting migratory destinations suggests that most leatherbacks returned to the same ocean region from where they originated prior to nesting. This apparent segregation of turtles to eastern and western Pacific locations provides evidence of foraging area philopatry among most leatherback turtles nesting in Indonesia. Although a novel concept for leatherbacks, philopatry to specific foraging regions illustrates the potential for within-population variation in environmental influences on reproduction and other life history traits. In light of the spatial mosaic of anthropogenic impacts occurring throughout the Pacific, this finding also suggests that segments of this nesting population experience differential impacts from human pressures.

The decline of Pacific leatherbacks has been attributed to egg harvest at nesting beaches [39], [60] and incidental capture (bycatch) in marine fisheries [61], [62]. Commercial fisheries are ubiquitous in both regions, although stringent fisheries management in U.S. waters of the eastern Pacific–which includes a special leatherback management zone (i.e. Leatherback critical habitat designation; [63])– compared to the more widespread presence of unregulated artisanal net and longline fisheries in the western Pacific suggest that these two areas present strongly different conservation challenges for leatherbacks. By establishing the foraging region for a large cross section of the nesting population of leatherbacks, we are able to separate potential differential influences affecting different portions of the population. Further, by exploring the relative proportion of putative eastern vs. western Pacific foragers over larger time scales, discrepancies in return migration rates (i.e. survivorship) between the foraging groups may be revealed and perhaps will allow for the design of more spatially appropriate management recommendations.

## Materials and Methods

### Study Site

This research was conducted at Jamursba-Medi, a 18-km long nesting beach along the northern Bird’s Head coast in Papua Barat Indonesia (0° 22’ S, 132° 33’ E). This nesting beach hosts the largest remaining nesting aggregation for leatherback turtles in the Pacific Ocean, and has been the site of extensive monitoring and research efforts for more than a decade [64]. The nesting season extends from April to September, peaking in July [64].

### Tissue Sampling and Preparation

Epidermis skin samples (i.e., stratum corneum; 0.10–0.25 g wet mass) were collected from 78 leatherbacks in 2005, 2006, 2007, and 2010 upon each turtle’s first witnessed nesting at the beginning of the nesting season with a razor from the dorsal surface of a hind flipper and stored in NaCl solution until transport to the lab. The top layer of the skin (stratum corneum) was separated from the underlying tissue (stratum germinativum), rinsed with deionized water, finely diced with a scalpel blade, then freeze-dried at –50°C for 12 h in a lyophilizer (BenchTop K, VirTis, SP Industries, Gardiner, NY, USA). Lipids were removed from skin samples using an accelerated solvent extractor (Model 200, Dionex, Bannockburn, IL, USA) with petroleum ether as the primary solvent. Following lipid extraction the samples were freeze dried at –50°C for 3 h to remove any residual solvent. Sub-samples of prepared tissue (0.6–1.0 mg) were weighed with a microbalance and packed in tin capsules for mass spectrometric analysis.

### Bulk Skin Stable Isotope Analysis

Bulk skin δ^13^C and δ^15^N stable isotopic compositions were determined using an on-line C-N analyzer coupled with an isotope ratio mass spectrometer (Finnigan ConFlo II/Delta-Plus). Approximately 1.0 mg of each sample was loaded into sterilized tin capsules and analyzed by a continuous-flow isotope-ratio mass spectrometer in the Stable Isotope Laboratory at Scripps Institution of Oceanography, La Jolla, California, USA. We used a Costech ECS 4010 elemental combustion system interfaced via a ConFlo III device (Finnigan MAT, Bremen, Germany) to a Deltaplus gas isotope-ratio mass spectrometer (Finnigan MAT, Bremen, Germany). Sample stable isotope ratios relative to the isotope standard are expressed in the following conventional delta (δ) notation in parts per thousand (‰):
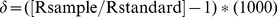
(1)where R_sample_ and R_standard_ are the corresponding ratios of heavy to light isotopes (e.g.^15^N/^14^N) in the sample and standard, respectively. R_standard_ for ^13^C was Baker Acetanilide (C_8_H_9_NO; δ^13^C = –10.4) calibrated monthly against the Peedee Belemnite (PDB) limestone formation international standard. R_standard_ for ^15^N was IAEA N1 Ammonium Sulfate ((NH_4_)_2_SO_4_; δ^15^N = +0.4) calibrated monthly against atmospheric N_2_ and USGS Nitrogen standards. All analytical runs included samples of standard materials inserted every 6 to 7 samples to calibrate the system and compensate for any drift over time. Hundreds of replicate assays of reference materials indicated measurement errors of 0.06‰ and 0.12‰ for carbon and nitrogen, respectively. Samples were combusted in pure oxygen in the elemental analyzer. Resultant gasses were reduced to N_2_ and CO_2_ gasses and passed through a series of thermal conductivity detectors and element traps to determine percent compositions. Acetanilide (71.09% C; 10.36% N) was used for calibration.

### Satellite Telemetry

Deployments were concentrated after the peak of each nesting season (*n*_2007_ = 12, *n*_2010_ = 1) to maximize the amount of post-nesting movement data. We used a combination of satellite-linked transmitters on leatherback turtles during the study period (Table S1), including the following platform transmitter terminals (PTT) models: Wildlife Computers (Washington, USA) MK10 (*n* = 1) and SPLASH tags (*n* = 1); Telonics (Arizona, USA) ST20 (*n* = 4); and Sea Mammal Research Unit (SMRU, U.K.) Satellite Relay Data Loggers (SRDL) (*n* = 7). All PTTs featured a salt-water switch that suppressed transmission while submerged and were duty-cycled to optimize battery life (6 h on, 19 h off), reporting positions via System Argos (Landover, Maryland) every 1–3 d. Only the best position for each day with an Argos location quality code of ‘LC0’ or better (between 250 m and >1.5 km accuracy) was included in the tracks.

Before attaching transmitters we visually assessed each turtle for signs of injury or compromised health and only selected turtles that appeared to be in normal condition. To minimize impacts on nesting females, we waited until turtles began laying eggs, well into the nesting process when females enter a trance-like state, before sampling and attaching the transmitters. During handling we measured curved carapace length (CCL) and width (CCW), applied PIT and flipper tags [65], and attached the transmitters.

The 13 turtles tracked in this study include the 12 turtles tracked in 2007 [40] for which skin samples were available, and an additional tissue-sampled leatherback tracked in 2010. In 2007, we attached PTTs to leatherback turtles see 40 with the aid of a flexible harness that consisted of soft nylon webbing with flexible polyvinyl tubing over the shoulder straps and a corrodible pin designed to release the harness within 1824 months 40. In 2010, the single transmitter was attached to the nesting female with a direct attachment approach [see 66].

### Compound Specific Stable Isotope Analysis of Amino Acids (CSIA-AA)

Skin samples from six satellite-tracked turtles (three that moved to the eastern Pacific and three that moved to the western Pacific) were analyzed for δ^15^N analysis of amino acids (Table S2). Although this technique is more expensive than δ^15^N analysis of bulk tissue, careful sample selection can yield important information that will enhance understanding of bulk isotope data sampled from a larger sample pool.

Samples were prepared for CSIA-AA by acid hydrolysis followed by derivatization to produce trifluoroacetic amino acid esters [67] using standard methods [41], [68]. Stable nitrogen isotopic composition was determined using a Delta XP mass spectrometer interfaced with a Trace Gas Chromatograph [68]. Measured isotopic compositions were corrected relative to known values for internal reference material - norleucine and aminoadipic acid. Samples were run in triplicate and standard deviations for each sample averaged 0.4‰ (range: <0.1 to 2.6‰). Average δ^15^N for source and trophic AAs was calculated with S.D. as follows:
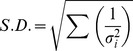
(2)


To confirm consistency in the δ^15^N measurement accuracy between the Scripps Institution of Oceanography mass spectrometer (used for bulk skin analyses) with that from the mass spectrometer at University of Hawaii we conducted bulk analyses on the same tissue samples as those from six individuals. These samples were analyzed for bulk carbon and nitrogen stable isotope composition, and the nitrogen isotopic composition of specific amino acids (Table S2).

### Calculation of Trophic Position

The fractional trophic positions of leatherback samples were calculated using the measured δ^15^N values of glutamic acid and phenylalanine [Eq. 3; 26]. There are two assumed factors when using AAs to calculate trophic position - the trophic enrichment factor (TEF), and the so-called Beta value (β). The TEF is the difference between glutamic acid and phenylalanine at each trophic step (+7.6‰) and the β value (+3.4‰) is the difference between glutamic acid and phenylalanine δ^15^N values in marine plants [26]. Error associated with the trophic position calculation was determined by propagation of error using the uncertainly in β and the trophic enrichment factor [26] and the measured reproducibility for glutamic acid and phenylalanine for each sample.

The nitrogen isotopic composition of AAs from a wide range of marine primary producers, herbivores and omnivores has been derived with an equation using the isotopic composition of glutamic acid and phenylalanine to estimate fractional trophic position in the consumers [Eq. 3; 26]. The relationship between the δ^15^N values of glutamic acid and phenylalanine has been found to provide a good indicator of trophic position (TP_AA_) in a range of species including various zooplankton and fish [26], [68], but has yet to be tested in marine reptiles such as turtles.
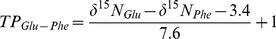
(3)


### Foraging Region Assignment

To model the difference in isotope values between the two foraging grounds, we conducted a Bayesian mixture-model analysis [69, pp. 220–223]. We assumed that leatherback turtles from the nesting beach had come from one of the two foraging regions that have different isotopic characteristics, and that isotope values in turtle tissues could be modeled with normal distributions using different means for each foraging region. Turtles were treated as independent samples. We looked at two basic models for the underlying isotopic distributions for the two foraging grounds: (1) two means and a common variance and (2) two means and two variances.

Isotope samples from turtles with telemetry data were treated in two ways: (1) without using the known foraging destinations from satellite telemetry results, and (2) using the foraging ground destinations. The proportions of turtles that use the two foraging grounds were estimated as either time independent or dependent. When they are treated as time dependent, these annual proportions were treated as independent of each other.

With the treatment of means and variances, telemetry data, and proportions of foraging grounds, a total of six models were fitted to the data (Table 3). Appropriateness of models was examined using posterior simulation, in which the observed data were compared to observable (or simulated) data [70]. We simulated a dataset of the same size for each set of parameters from the joint posterior distribution. Both the observed and each simulated dataset were sorted from the smallest to largest values and the sum of the differences between the datasets computed. The distribution of sums of differences was used to determine the model fit. OpenBugs (version 3.2.1) [71] was used to obtain the joint posterior distribution of the parameters through Markov chain Monte Carlo. Four independent chains of 105,000 steps with randomized starting points were used to sample from the joint posterior distribution. The first 5000 steps were used to tune the sampling algorithm and discarded for computing summary statistics. For the remaining 100,000 samples of each chain, every five steps were retained to reduce the serial autocorrelations. Posterior samples of 80,000, therefore, were used to compute the summary statistics for the means and variance of normal distributions of the stable isotope values for the two foraging grounds, the proportion of turtles that belong to each foraging ground, and the assignment of each turtle to a foraging ground with associated probability. For all parameters, we used vague prior distributions within appropriate parameter spaces. To make an inference about the proportion of turtles that forage in the eastern or western Pacific Ocean, we arbitrarily defined the probability 0.8 as the cut-off point.

## Supporting Information

Table S1 Summary of satellite transmitter deployments and body size information for leatherback turtles nesting at Jamursba-Medi, Papua Barat, Indonesia.
(DOC)

Table S2 Bulk δ^15^N and δ^13^C and compound specific δ^15^N (CSIA-AA) values for glutamic acid (Glu) and phenylalanine (Phe) from leatherback turtle skin samples collected in Jambursba Medi, Indonesia.
(DOC)
