# Supplementary material for: Stable Isotope Tracking of Endangered Sea Turtles: Validation with Satellite Telemetry and δ15N Analysis of Amino Acids
Source: PLoS One. 2012 May 29;7(5):e37403. doi: 10.1371/journal.pone.0037403 (PMC3362573; doi:10.1371/journal.pone.0037403)
Supplement: Table S1 — Summary of satellite transmitter deployments and body size information for leatherback turtles nesting at Jamursba-Medi, Papua Barat, Indonesia. (DOC) [file pone.0037403.s001.doc]

**Table S1.** Summary of satellite transmitter deployments and body size information for leatherback turtles nesting at Jamursba Medi, Papua Barat, Indonesia.

| **Date** | **PTT ID** | **PTT Type** | **Track Duration**  **(days)** | **Foraging Destination** | **Skin**  **δ^15^N** | **CCL** | **CCW** |
| --- | --- | --- | --- | --- | --- | --- | --- |
| 22/06/2007 | 72491 | SMRU-SRDL | 271 | EP | 15.45 | 137* | 114 |
| 22/06/2007 | 72488 | SMRU-SRDL | 357 | WP | 10.85 | 153 | 105 |
| 22/06/2007 | 72487 | SMRU-SRDL | 364 | WP | 10.63 | 150 | 111 |
| 23/06/2007 | 72486 | SMRU-SRDL | 289 | EP | 15.21 | 169 | 124 |
| 23/06/2007 | 72484 | SMRU-SRDL | 245 | WP | 11.11 | 166 | 118 |
| 23/06/2007 | 24646 | Telonics ST-20 | 429 | WP | 10.87 | 159 | 110 |
| 26/06/2007 | 23662 | Telonics ST-20 | 383 | EP | 17.35 | 163 | 120 |
| 26/06/2007 | 40671 | Telonics ST-20 | 519 | EP | 15.94 | 165 | 115 |
| 26/06/2007 | 40673 | Telonics ST-20 | 304 | WP | 11.58 | 149 | 91 |
| 27/06/2007 | 61714 | SMRU-SRDL | 46 | WP | 10.84 | 161 | 114 |
| 27/06/2007 | 72489 | SMRU-SRDL | 434 | WP | 15.05 | 161 | 118 |
| 29/06/2007 | 23660 | WC-SPLASH | 95 | WP | 11.43 | 152 | 111 |
| 09/06/2010 | 78500 | WC-MK10 | 324 | EP | 15.29 | 163 | 118 |

All but the 2010 deployment (direct attachment) were deployed using the harness method (Benson et al. 2011). PTT, Platform Terminal Transponder (satellite transmitter); CCL, curved carapace length; CCW, curved carapace wideth; EP, eastern Pacific; WP, western Pacific; *, leatherback turtle missing part of pygal attachment.
