# Supplementary material for: Stable Isotope Tracking of Endangered Sea Turtles: Validation with Satellite Telemetry and δ15N Analysis of Amino Acids
Source: PLoS One. 2012 May 29;7(5):e37403. doi: 10.1371/journal.pone.0037403 (PMC3362573; doi:10.1371/journal.pone.0037403)
Supplement: Table S2 — Bulk δ15N and δ13C and compound specific δ15N (CSIA-AA) values for glutamic acid (Glu) and phenylalanine (Phe) from leatherback turtle skin samples collected in Jambursba Medi, Indonesia. (DOC) [file pone.0037403.s002.doc]

**Table S2.** Bulk δ^15^N and δ^13^C and compound specific δ^15^N (CSIA-AA) values for glutamic acid (Glu) and phenylalanine (Phe) from leatherback turtle skin samples collected in Jambursba Medi, Indonesia.

|  |  | **Bulk tissue (‰)** | | | | **CSIA-AA (‰)** | | **Trophic** |
| --- | --- | --- | --- | --- | --- | --- | --- | --- |
|  |  | **NOAA** | | **UH** | | **Phe** | **Glu** | **Position** |
| **Turtle** | **Destination** | **δ^15^N** | **δ^13^C** | **δ^15^N** | **δ^13^C** | **Av (SD)** | **Av (SD)** | **TP_AA_ (SD)** |
| 1 | WP | 10.85 | –16.71 | 10.54 | –15.24 | 4.86 (0.71) | 17.44 (0.36) | 2.21 (0.20) |
| 2 | WP | 10.84 | –17.16 | 11.28 | –15.65 | 3.57 (0.52) | 17.41 (0.26) | 2.37 (0.18) |
| 3 | WP | 10.63 | –17.16 | 10.79 | –15.86 | 4.02 (0.26) | 18.12 (0.20) | 2.41 (0.14) |
| 4 | EP | 15.45 | –17.90 | 15.74 | –16.78 | 7.37 (0.36) | 21.30 (0.60) | 2.39 (0.20) |
| 5 | EP | 15.94 | –17.51 | 16.22 | –16.50 | 6.90 (0.26) | 20.17 (0.71) | 2.30 (0.20) |
| 6 | EP | 15.21 | –17.08 | 14.74 | –16.08 | 7.46 (0.39) | 21.43 (0.26) | 2.39 (0.16) |

Sample results are presented for samples analyzed at NOAA-Southwest Fisheries Science Center (NOAA) and University of Hawaii (UH). Turtles were equipped with satellite transmitters after sampling and were tracked to foraging areas in the western Pacific (WP) and eastern Pacific (EP)(Fig.1).
